# Specific Dietary (Poly)phenols Are Associated with Sleep Quality in a Cohort of Italian Adults

**DOI:** 10.3390/nu12051226

**Published:** 2020-04-26

**Authors:** Justyna Godos, Raffaele Ferri, Sabrina Castellano, Donato Angelino, Pedro Mena, Daniele Del Rio, Filippo Caraci, Fabio Galvano, Giuseppe Grosso

**Affiliations:** 1 Oasi Research Institute—IRCCS, 94018 Troina, Italy; justyna.godos@gmail.com (J.G.); rferri@oasi.en.it (R.F.); carafil@hotmail.com (F.C.); 2 Department of Educational Sciences, University of Catania, 95124 Catania, Italy; sabrina.castellano@unict.it; 3 Faculty of Bioscience and Technology for Food, Agriculture and Environment, University of Teramo, 64100 Teramo, Italy; dangelino@unite.it; 4 Human Nutrition Unit, Department of Food and Drugs, University of Parma, 43125 Parma, Italy; pedromiguel.menaparreno@unipr.it; 5 School of Advanced Studies on Food and Nutrition, University of Parma, 43125 Parma, Italy; daniele.delrio@unipr.it; 6 Department of Veterinary Medicine, University of Parma, 43125 Parma, Italy; 7 Department of Drug Sciences, University of Catania, 95125 Catania, Italy; 8 Department of Biomedical and Biotechnological Sciences, University of Catania, 95123 Catania, Italy; fgalvano@unict.it

**Keywords:** polyphenol, sleep, mental health, cohort, antioxidant, cognitive, brain, Sicily, population

## Abstract

Background: Diet has been the major focus of attention as a leading risk factor for non-communicable diseases, including mental health disorders. A large body of literature supports the hypothesis that there is a bidirectional association between sleep and diet quality, possibly via the modulation of neuro-inflammation, adult neurogenesis and synaptic and neuronal plasticity. In the present study, the association between dietary total, subclasses of and individual (poly)phenols and sleep quality was explored in a cohort of Italian adults. Methods: The demographic and dietary characteristics of 1936 adults living in southern Italy were analyzed. Food frequency questionnaires (FFQs) were used to assess dietary intake. Data on the (poly)phenol content in foods were retrieved from the Phenol-Explorer database. The Pittsburg Sleep Quality Index was used to measure sleep quality. Multivariate logistic regression analyses were used to test the associations. Results: A significant inverse association between a higher dietary intake of lignans and inadequate sleep quality was found. Additionally, individuals with the highest quartile of hydroxycinnamic acid intake were less likely to have inadequate sleep quality. When individual compounds were taken into consideration, an association with sleep quality was observed for naringenin and apigenin among flavonoids, and for matairesinol among lignans. A secondary analysis was conducted, stratifying the population into normal weight and overweight/obese individuals. The findings in normal weight individuals showed a stronger association between certain classes of, subclasses of and individual compounds and sleep quality. Notably, nearly all individual compounds belonging to the lignan class were inversely associated with inadequate sleep quality. In the overweight/obese individuals, there were no associations between any dietary (poly)phenol class and sleep quality. Conclusions: The results of this study suggest that a higher dietary intake of certain (poly)phenols may be associated with better sleep quality among adult individuals.

## 1. Introduction

Diet has been the focus of major attention as a leading risk factor for non-communicable diseases [1,2]. These estimates are based on convincing evidence that dietary factors may play a role in the risks of cardiovascular diseases and certain cancers [3,4,5,6,7,8]. A more intriguing hypothesis recently explored is that diet may also influence brain health and mental disorders [9,10]. A large body of literature supports the hypothesis that there is an association between sleeping patterns and diet quality, possibly mediating weight status and obesity-related disorders [11]. Generally, most of the evidence relies on the positive association between sleep quality or duration and diet quality, but relatively recent studies suggest that a bidirectional relationship may exist, with dietary factors influencing sleep features [12]. Several mechanisms have been hypothesized to explain this association, including inflammation, oxidative stress, the gut microbiome, epigenetic modifications and the direct effects of nutrients and non-nutrients on neuroplasticity [13]. Among the healthy dietary patterns suggested for their putative influence on sleep quality, plant-based foods, including vegetables, grains, nuts, seeds, legumes and fruits, have demonstrated to have a mechanistic relationship with better mental health, potentially influencing sleep features [12]. Those foods are rich sources of bioactive compounds that, in the context of a healthy lifestyle, may play a potential role in preventing subclinical low-grade inflammation, a starting point for several chronic non-communicable diseases as well as for impaired sleep quality and duration [14,15]. There is, in fact, evidence that inflammation may mediate a variety of brain disorders involving sleep quality but also stress, depression, dementia and Alzheimer’s disease [16]. Thus, it is crucial to understand whether diet may affect the level of inflammation and which compounds should be of major interest.

Dietary (poly)phenols represent a group of compounds present in plant-derived foods that, based on their biochemical structure, may play a pivotal role in radical scavenging and in mediating inflammation processes [17,18,19]. Several families are commonly consumed when adhering to healthy dietary patterns, including flavonoids (mostly contained in fruits, vegetables, tea and cocoa products), phenolic acids (contained in fruits, coffee, pulses and nuts), stilbenes (mainly contained in wine) and phytoestrogens (including isoflavones and lignans, contained in soy products and legumes). Dietary (poly)phenols have been related to several potential health benefits [20]: recently, they have been hypothesized to also play a role in brain health [21,22]. We previously reported that individuals more adherent to healthy dietary patterns (i.e., the Mediterranean diet) and to a diet with low inflammatory potential were more likely to have higher sleep quality [23,24]. In the present study, we aimed to test whether total, subclasses of and individual (poly)phenols may be candidate molecules associated with sleep quality in a cohort of Italian adults.

## 2. Materials and Methods

### 2.1. Study Population 

The MEAL study is an observational study aiming to investigate the association between the nutritional and lifestyle habits characterizing the classical Mediterranean area and non-communicable diseases. The baseline data included a sample of 2044 men and women aged 18 or more years old. Individuals were randomly selected in the main districts of the city of Catania, Sicily, Italy. The enrolment and data collection were performed between 2014 and 2015. Details of the study protocol are published elsewhere [25]. All participants were informed about the aims of the study and provided written informed consent. All the study procedures were carried out in accordance with the Declaration of Helsinki (1989) of the World Medical Association. The study protocol has been reviewed and approved by the concerning ethical committee of the Municipal Health Authority (protocol number: 802/23 December 2014).

### 2.2. Data Collection

Electronic data collection was performed by face-to-face assisted personal interviews, using tablet computers. In order to visualize the response options, participants were provided with a paper copy of the questionnaire. However, final answers were registered directly by the interviewer. The demographic data included gender, age at recruitment, highest educational degree achieved, occupation (specifying the nature of the most important employment during the year before the investigation) or last occupation before retirement, and marital status. Educational status was categorized as (i) low (primary/secondary), (ii) medium (high school), and (iii) high (university). Occupational status was categorized as (i) unemployed, (ii) low (unskilled workers), (iii) medium (partially skilled workers), and (iv) high (skilled workers). Physical activity status was evaluated using International Physical Activity Questionnaires (IPAQ) [26], which demonstrated an acceptable validity for the Italian population (the Cronbach’s alpha values were 0.73 and 0.60 for the short and long versions, respectively) [27]: the instrument included a set of questionnaires (five domains) investigating the time spent being physically active in the last 7 days. Based on the IPAQ guidelines, the final score allows categorizing physical activity levels as (i) low, (ii) moderate, and (iii) high. Smoking status was categorized as (i) non-smoker, (ii) ex-smoker, and (iii) current smoker. Alcohol consumption was categorized as (i) none, (ii) moderate drinker (0.1–12 g/d) and (iii) regular drinker (>12 g/d). Anthropometric measurements have been collected following standard procedures [28]. Arterial blood pressure was measured in sitting position and after at least 5 min of rest, at the end of the physical examination. Because of the possibility of differences in blood pressure measurements, the measurements were taken three times at the right arm, relaxed and well supported by a table, with an angle of 45° from the trunk. A mean of the last two measurements was considered for inclusion in the database. Patients were considered hypertensive when their average systolic/diastolic blood pressure levels were higher than or equal to 140/90 mm Hg, they were taking anti-hypertensive medications, or they had previously been diagnosed with hypertension.

### 2.3. Dietary Assessment

The dietary assessment was performed by the administration of two food frequency questionnaires (FFQ, long and short versions) that had been previously tested for validity and reliability for individuals living in Sicily [29,30]. For the purposes of this study, the data from the most comprehensive FFQ including 110 food items were used. The identification of food intake, energy content and macro- and micro-nutrient intake was performed through comparison with the food composition tables from the Research Center for Foods and Nutrition [31]. The intake of seasonal foods referred to consumption during the period in which the food was available, proportionally adjusted by its intake in one year. The instrument showed a good relative validity (all major food groups with the exception of bread and soft drinks had significant Person’s correlation coefficients, over 0.60, and the highest correlation coefficients for coffee (*R* = 0.96 in men and women), tea (*R* = 0.79 in men and 0.80 in women) and alcoholic beverages (*R* = 0.83 in men and 0.88 in women)) and reliability (all major food groups besides bread had significant Person’s correlation coefficients, over 0.60, and the highest correlation coefficients for coffee (*R* = 0.97 in men and *R* = 0.96 in women), tea (*R* = 0.82 in men and 0.84 in women) and alcoholic beverages (*R* = 0.87 in men and 0.92 in women)). FFQs with unreliable intakes (<1000 or >6000 kcal/d) were excluded from the analyses (*n* = 107), leaving a total of 1936 individuals included in the analysis.

### 2.4. Estimation of Polyphenol Intake

The process of the estimation of habitual (poly)phenol intakes has been previously described in detail [32]. Briefly, data on the (poly)phenol content in foods were retrieved from the Phenol-Explorer database (www.phenol-explorer.eu) [33]. A new version of the Phenol-Explorer database containing data on the effects of cooking and food processing on (poly)phenol contents was used whenever possible, in order to apply (poly)phenol-specific retention factors [34]. Foods that contained no (poly)phenols were excluded from the calculation, leaving a total of 75 items included in the analyses. Food weight loss or gain during cooking was corrected using yield factors [35]. Average food consumption was calculated (in g or mL) by following the standard portion sizes used in the study and then converted to 24 h intake. Finally, a search was carried out in the Phenol-Explorer database to retrieve the mean content values for all (poly)phenols contained in the selected foods. Next, (poly)phenol intake from each food was calculated by multiplying the content of each (poly)phenol class by the daily consumption of each food. The total (poly)phenol intake was considered as the sum of all the main classes and subclasses. Finally, (poly)phenol intake was adjusted for total energy intake (kcal/d) using the residual method [36].

### 2.5. Sleep Quality

The Pittsburg sleep quality index (PSQI) [37] was used to assess participants’ sleep quality and disturbances in the past six months, which has also been demonstrated to be a good and reliable tool in the Italian population (the internal consistency was represented by a Cronbach’s alpha of 0.835) [38]. It consists of 19 items that are rated on a four-point scale (0–3) and grouped into seven components (sleep quality, sleep latency, sleep duration, habitual sleep efficiency, sleep disturbance, the use of sleeping medications, and daytime dysfunction). The item scores in each component were summed and converted to component scores ranging from 0 (better) to 3 (worse) based on guidelines. The total PSQI score was calculated as the summation of seven component scores, ranging from 0 to 21, where a higher score indicates a worse condition. A score of <5 on for total global PSQI is indicative of adequate sleep quality.

### 2.6. Statistical Analysis

Frequencies are expressed as absolute numbers and percentages; continuous variables are expressed as means and standard deviations. Individuals were divided into quartiles of dietary (poly)phenol intake and the distributions of background characteristics were compared between the groups. Differences were tested with Chi-square tests for categorical variables, ANOVA for continuous variables distributed normally, and Kruskall–Wallis tests for variables not normally distributed. Energy-adjusted multivariate logistic regression models were used to test the association between the variables of exposure (including total (poly)phenols, their classes, subclasses and individual compounds) and inadequate sleep quality; a multivariate model adjusted for all other background characteristics (body mass index, physical activity, educational status, occupational status, smoking status, alcohol consumption, occurrence of hypertension, diabetes, dyslipidemias, cardiovascular disease, cancer, and menopausal status) was also used to test whether the observed associations were independent from the aforementioned variables. All reported *P*-values were based on two-sided tests and compared to a significance level of 5%. Bonferroni correction was applied and *P*-values meeting the threshold of 0.05 divided by the number of polyphenol quartiles were noted. The SPSS 17 (SPSS Inc., Chicago, IL, USA) software was used for all the statistical calculations.

## 3. Results

The baseline characteristics of the study population by quartiles of energy-adjusted total (poly)phenol intake are presented in Table 1. The distributions of certain variables, such as age and education level, did not follow linear trends, as individuals in the middle quartiles were significantly older and had lower educational levels than the others. The participants in the highest quartile of total (poly)phenol intake had moderate levels of physical activity and were moderate or regular alcohol drinkers, and they also had a lower prevalence of hypertension, while concerning type 2 diabetes, the distribution was not linear and higher rates were registered in the middle quartiles (Table 1). High total (poly)phenol intake was also correlated with higher total energy intake (Table 1).

A total of 509 individuals (32.4%) reported inadequate sleep quality. No association between total or individual major classes of (poly)phenols and sleep quality was found, with the exception of lignans, for which participants in the third quartile of intake were less likely to have inadequate sleep quality after adjusting for potential confounding factors (OR = 0.62; 95% CI: 0.43, 0.88; Table 2). None of the flavonoid subclasses showed an association with sleep quality. Conversely, individuals with the highest quartile of hydroxycinnamic acid intake were less likely to have inadequate sleep quality (OR = 0.67; 95% CI: 0.46, 0.98; Table 2). When individual compounds were taken into consideration, inverse associations with inadequate sleep quality were observed for naringenin (OR = 0.66; 95% CI: 0.46, 0.95) and apigenin (OR = 0.63; 95% CI: 0.44, 0.90) among flavonoids, and for matairesinol (OR= 0.66; 95% CI: 0.46, 0.96) among lignans (Table 2).

A secondary analysis was conducted, stratifying the population into normal weight and overweight/obese individuals. The findings in normal weight individuals showed a stronger association between certain classes, subclasses and individual compounds and sleep quality (Table 3). Notably, nearly all individual compounds belonging to the lignan class (secoisolariciresinol, matairesinol and pinoresinol) were inversely associated with inadequate sleep quality (Table 3).

To the contrary, in the overweight/obese individuals, there were no associations between any dietary (poly)phenol class and sleep quality. Among the individual components, only apigenin was significantly associated with sleep quality (OR = 0.53; 95% CI: 0.31, 0.90) (Table 4).

## 4. Discussion

In this article, we tested whether dietary (poly)phenols were associated with sleep quality in a cohort of Italian adults. Individuals showing a higher intake of some flavonoid subclasses (flavanones and flavones), phenolic acids (such as hydroxycinnamic acids) and lignans were significantly less likely to have inadequate sleep quality. These findings suggest that some classes of (poly)phenol may play a specific role when exploring their relationship with brain and mental health. Interestingly, the associations were more evident when stratifying the cohort by weight status, showing significant results in normal weight individuals, but no confirmed associations for overweight/obese participants.

To date, only one recent study has investigated the relationship between dietary (poly)phenols derived from fruit and vegetables and sleep duration [39]. The study was conducted on 13,958 women with about 4 years of follow-up in the UK Women’s Cohort Study: total fruit and vegetable consumption and their estimated content of total polyphenols were directly associated with sleep duration, while individual (poly)phenol classes were not associated with the outcome of interest [39]. Despite no other studies being focused on polyphenols, some studies reported a direct relationship between sleep duration and quality, and fruit and vegetable intake [40,41]. Other studies showed the role of certain polyphenol-rich foods (i.e., black tea and cocoa products) in improving sleep quality [42,43]. Despite there being no other studies specifically conducted on (poly)phenols and sleep quality, there is consistent evidence from the literature suggesting a potential role of dietary (poly)phenols in improving mental health and preventing conditions that are associated with sleep disorders. For instance, some cohort studies showed that individuals with higher intakes of the same flavonoid, hydroxycinnamic and lignan classes found to be significantly associated with better sleep quality in this study were less likely to have depressive symptoms [44,45]. Other studies also showed an inverse association between fruit and vegetable intake and depressive symptoms and perceived stress, despite most of them have been conducted on students [46,47,48,49].

From a general mechanistic point of view, (poly)phenol circulating metabolites are able to pass through the blood–brain barrier to various extents, depending on their degree of lipophilicity, with less polar (poly)phenol metabolites capable of greater brain uptake than more polar ones [50,51]. The main potential beneficial effects of dietary (poly)phenols in the central nervous system include the suppression of neuronal apoptosis, modulation of signaling pathways implicated in neuron survival, and stimulation of adult neurogenesis [52,53,54]. With special regard to specific mechanisms related to sleep features, dietary (poly)phenols have been shown to improve resilience after sleep deprivation [55]; some individual molecules, such as apigenin, are able to reduce locomotor activity, prolong sleep time, increase sleep rate increase and sleep time in combined administration with a GABA(A) receptor agonist, and show synergic effects in potentiating sleep onset in animal models [56,57,58]. Additionally, derivates of hydroxycinnamic acids have been identified as agonists for both gamma-amino butyric acid (GABA) receptors and act synergistically with 5-hydroxytryptophan (5-HTP), both of which play a role in sleep quality, including having sedative effects on locomotion activity, prolonging sleeping time and shortening sleep latency [59,60,61]. 

Dietary (poly)phenols have been shown to decrease systemic inflammation [62] but also exert anti-neuroinflammatory properties and reduce oxidative stress and inflammation-related conditions [63]. Several studies have shown that molecules of interest from our study, including some flavonoids (i.e., apigenin) and hydroxycinnamic acids, improve cell antioxidant activity against oxidative stress in the central nervous system [64,65]. Additionally, lignans have been demonstrated to exert anti-oxidative and anti-inflammatory properties in neurons and protect the blood–brain barrier against inflammatory cells by reducing oxidative stress, inflammation and permeability [66,67,68]. Dietary (poly)phenols may ameliorate poor endothelial function [69] and help to control blood pressure [70], which, in turn, has been associated with measures of sleep quality together with decreases in the percentage of REM sleep and increases in REM sleep latency [71,72,73]. Previous epidemiological studies have shown an inverse association between the intake of specific classes of (poly)phenol (in line with the findings shown in the present study)—including flavones and flavanones among flavonoids [74], and hydroxycinnamic acids—and the occurrence of hypertension [75,76]. The mechanisms underlying these relationships are still under investigation; besides, regarding the direct effect of (poly)phenols (especially hydroxycinnamic acids) on low-grade inflammation, which, in turn, may affect endothelial function [77,78], an intriguing hypothesis involves nitric oxide-mediated vasodilation in the brain, which has been shown to facilitate REM sleep [79].

An emerging body of literature investigates the double interexchange of information between the gut microbiota and the brain through a complex system of signals involving neural, endocrine and inflammatory mechanisms [80]. In fact, the gut microbiota has been shown to affect brain and behaviors related to anxiety and depressive symptoms depending on bacterial family ratios, dysbiosis, and subsequent modulation through dietary (poly)phenol intake [81]; the status of the pro- and anti-inflammatory balance in the gut has been demonstrated to have an impact at the systemic level and on the central nervous system [82,83]. Recent studies show that dietary (poly)phenols may play a role in the modulation of gut microbiota metabolism and that variations in the gut microbiota can affect (poly)phenol activity [84]. This hypothesis is particularly valid in light of our results stratified by weight status; in the intestinal microbiota of obese people, a specific increase in the proportion of class Firmicutes to class Bacteroidetes has been shown compared to in normal-weight individuals [85], which may affect (poly)phenol transformation and absorption in the gut [86] and their anti-oxidant effects [87,88,89]. However, current evidence is still limited, largely based on cell and animal studies, and future studies conducted on humans are needed to identify specific metabotypes associated with activity in the brain.

To the best of our knowledge, this is the first study investigating such a comprehensive group of compounds in order to identify key (poly)phenol molecules of potential interest to improve sleep quality. Moreover, based on our previous results [32], no unique food source is responsible for specific compounds or classes of (poly)phenols; thus, the present analysis is able to detect the potential role of (poly)phenols rather than of the individual foods underlying their consumption. However, the findings presented in this study should be considered in light of some limitations. Firstly, this study provided evidence from a cross-sectional analysis, which cannot exclude reverse causation nor describe a causal relation. Secondly, all methods used to assess food consumption and dietary polyphenol intake provide only estimations, while true intake cannot be estimated without measuring biomarkers or metabolites. Despite the use of the Phenol-Explorer database being validated and widespread, this method cannot take into account molecular transformation or interaction. Moreover, recall bias and unmeasured confounding factors (i.e., jobs requiring night shifts) should be considered as potential limitations. However, these methods are commonly used in the current scientific literature, representing the standard for scientific research until new methods are validated and made available. Thirdly, no other aspects related to sleeping problems or other mental health issues have been considered, while they may be associated with sleep quality. Thus, the potential mediating effect of such intermediary conditions should be taken into account.

## 5. Conclusions

Our study suggests that a higher dietary intake of certain (poly)phenols may be potentially associated with better sleep quality. However, further epidemiological studies are needed to confirm the presented hypothesis, with a major focus on sleep quality. Several aspects should be further considered in future studies, such as the use of caffeinated beverages or the timing of food and alcohol intake. Future studies should additionally focus on the inter-individual variation in response to the consumption of (poly)phenols and thus investigate the associations not only for their dietary intake but also for the true internal exposure to their metabolites. In this context, attention to the gut microbiota composition should also be paid as differences in microbial species may condition (poly)phenol metabolite formation and bioactivity. Finally, intervention studies will be needed to explore the level of absorption and bioavailability of dietary (poly)phenols and the characterization of biologically available (poly)phenol metabolites responsible for the promotion of resilience against cognitive impairment in response to poor sleep quality.

## Figures and Tables

**Table 1:** Background characteristics of participants in the MEAL cohort by quartiles of total (poly)phenol intake (energy-adjusted).

	Total (Poly)phenol Intake				*P*
	Q1	Q2	Q3	Q4	
Age (years), mean (SD)	47.0 (19.3)	48.9 (18.0)	50.1 (16.7)	47.6 (16.4)	0.036
Men, *n* (%)	193 (43.0)	217 (43.4)	195 (39.0)	199 (40.9)	0.472
BMI, mean (SD)	25.9 (4.4)	25.9 (4.8)	25.9 (4.5)	25.5 (4.6)	0.393
Smoking status, *n* (%)					0.598
Current	96 (21.4)	127 (25.4)	130 (26.0)	112 (23.0)	
Former	62 (13.8)	71 (14.2)	75 (15.0)	68 (14.0)	
Never	291 (64.8)	302 (60.4)	295 (59.0)	307 (63.0)	
Educational level, *n* (%)					0.001
Low	147 (32.7)	185 (37.0)	187 (37.4)	178 (36.6)	
Medium	153 (34.1)	180 (36.0)	213 (42.6)	174 (35.7)	
High	149 (33.2)	135 (27.0)	100 (20.0)	135 (27.7)	
Occupational level, *n* (%)					0.046
Unemployed	90 (23.7)	115 (26.7)	131 (28.8)	125 (31.9)	
Low	64 (16.8)	66 (15.3)	74 (16.3)	62 (15.8)	
Medium	87 (22.9)	126 (29.2)	123 (27.0)	104 (26.5)	
High	139 (36.6)	124 (28.8)	127 (27.9)	101 (25.8)	
Physical activity level, *n* (%)					0.010
Low	82 (20.4)	92 (20.4)	69 (19.7)	86 (19.7)	
Medium	192 (47.8)	236 (52.2)	200 (45.8)	228 (52.2)	
High	128 (31.8)	124 (27.4)	168 (38.4)	123 (28.1)	
Alcohol consumption, *n* (%)					<0.001
None	125 (27.8)	118 (23.6)	74 (14.8)	58 (11.9)	
Moderate (0.1–12 g/d)	317 (70.6)	340 (67.9)	306 (61.2)	243 (49.9)	
Regular (>12 g/d)	7 (1.6)	43 (8.6)	120 (24.0)	186 (38.2)	
Health status, *n* (%)					
Hypertension	240 (53.5)	275 (54.9)	261 (52.2)	200 (41.1)	<0.001
Diabetes	21 (4.7)	50 (10.0)	41 (8.2)	34 (7.0)	0.018
Dyslipidemias	69 (15.4)	100 (20.0)	102 (20.4)	85 (17.5)	0.158
Cardiovascular disease	40 (9.1)	36 (7.4)	42 (8.7)	36 (7.6)	0.732
Cancer	17 (3.8)	18 (3.6)	18 (3.6)	25 (5.1)	0.556
Menopausal status (women only), *n* (%)	118 (45.4)	129 (44.6)	146 (46.9)	133 (44.5)	0.926
Total energy intake (kcal/d), mean (SD)	1749.9 (563.4)	1916.2 (552.7)	2062.8 (625.2)	2704.2 (1068.9)	<0.001

**Table 2:** Odds ratios (ORs) and 95% confidence intervals (CIs) for the associations between (poly)phenol intake (total, main classes, subclasses and individual compounds) and adequate sleep quality in the MEAL cohort.

	(Poly)phenol Quartiles, OR (95% CI)				
	Q1	Q2	Q3	Q4	*P* for Trend
**Total (poly)phenols**	1	1.12 (0.80, 1.58)	0.87 (0.61, 1.25)	1.04 (0.69, 1.55)	0.476
**Total flavonoids**	1	0.83 (0.58, 1.18)	0.92 (0.64, 1.31)	0.97 (0.66, 1.43)	0.948
*Flavanols*	1	0.90 (0.63, 1.28)	0.93 (0.66, 1.32)	1.25 (0.89, 1.77)	0.603
Catechins	1	0.81 (0.57, 1.15)	0.96 (0.67, 1.38)	1.05 (0.73, 1.50)	0.255
*Flavonols*	1	1.07 (0.68, 1.67)	0.90 (0.48, 1.68)	1.32 (0.59, 2.92)	0.645
Quercetin	1	1.44 (0.97, 2.11)	1.51 (1.04, 2.20)	1.33 (0.88, 2.01)	0.368
Kaempferol	1	0.72 (0.50, 1.02)	0.74 (0.53, 1.05)	0.91 (0.59, 1.41)	0.104
*Flavanones*	1	1.03 (0.72, 1.45)	0.75 (0.53, 1.06)	0.72 (0.50, 1.03)	0.263
Hesperetin	1	0.85 (0.60, 1.21)	0.96 (0.68, 1.36)	0.81 (0.58, 1.15)	0.249
Naringenin	1	0.76 (0.54, 1.08)	0.62 (0.44, 0.89)	0.66 (0.46, 0.95)	0.020
*Flavones*	1	1.03 (0.72, 1.45)	0.75 (0.53, 1.06)	0.72 (0.50, 1.03)	0.016
Apigenin	1	0.67 (0.46, 0.96)	0.61 (0.41, 0.90)	0.63 (0.44, 0.90)	0.046
Luteolin	1	1.09 (0.77, 1.53)	0.85 (0.60, 1.20)	0.84 (0.56, 1.15)	0.043
*Anthocyanins*	1	0.91 (0.63, 1.31)	0.97 (0.68, 1.37)	0.86 (0.59, 1.26)	0.459
*Isoflavones*	1	0.90 (0.64, 1.27)	0.95 (0.68, 1.33)	0.92 (0.65, 1.30)	0.356
Daidzein	1	0.90 (0.64, 1.27)	0.98 (0.70, 1.38)	0.89 (0.62, 1.27)	0.301
Genistein	1	0.90 (0.64, 1.27)	0.93 (0.66, 1.32)	0.93 (0.65, 1.32)	0.263
Biochanin A	1	0.75 (0.51, 1.10)	0.96 (0.66, 1.41)	1.07 (0.72, 1.60)	0.025
**Phenolic acids**	1	1.32 (0.93, 1.87)	1.31 (0.97, 1.87)	1.16 (0.81, 1.65)	0.457
*Hydroxybenzoic acids*	1	1.10 (0.80, 1.53)	1.07 (0.74, 1.53)	1.13 (0.79, 1.61)	0.852
Vanillic acid	1	1.00 (0.69, 1.44)	1.34 (0.93, 1.93)	1.27 (0.85, 1.89)	0.624
*Hydroxycinnamic acids*	1	0.89 (0.63, 1.26)	0.77 (0.51, 1.09)	0.67 (0.46, 0.98)	0.005 ^a^
Caffeic acid	1	0.95 (0.67, 1.36)	0.72 (0.50, 1.04)	0.88 (0.54, 1.43)	0.055
Cinnamic acid	1	1.02 (0.73, 1.42)	0.93 (0.65, 1.35)	1.09 (0.77, 1.52)	0.680
Ferulic acid	1	0.71 (0.50, 1.01)	0.94 (0.67, 1.31)	0.77 (0.53, 1.12)	0.299
**Stilbenes**	1	0.73 (0.50, 1.06)	0.83 (0.56, 1.24)	0.97 (0.60, 1.56)	0.828
**Lignans**	1	0.85 (0.60, 1.21)	0.62 (0.43, 0.88)	0.78 (0.54, 1.12)	0.051
Lariciresinol	1	0.90 (0.64, 1.27)	0.69 (0.49, 0.98)	0.85 (0.59, 1.22)	0.187
Matairesinol	1	0.72 (0.51, 1.02)	0.67 (0.47, 0.95)	0.66 (0.46, 0.96)	0.017
Pinoresinol	1	0.90 (0.64, 1.27)	0.64 (0.45, 0.92)	0.78 (0.54, 1.12)	0.090
Secoisolariciresinol	1	0.72 (0.51, 1.02)	0.68 (0.48, 0.98)	0.80 (0.55, 1.16)	0.187

Adjusted for total energy intake (continuous), body mass index (continuous), physical activity (low/medium/high), educational status (low/medium/high), occupational status (unemployed/low/medium/high), smoking status (current/former/never), alcohol consumption (none/moderate/regular), occurrence of hypertension, diabetes, dyslipidemias, cardiovascular disease, cancer (yes/no), and menopausal status (women only, yes/no). ^a^ *P*-value meeting threshold for Bonferroni correction.

**Table 3:** Odds ratios (ORs) and 95% confidence intervals (CIs) for the associations between (poly)phenol intake (total, main classes, subclasses and individual compounds) and adequate sleep quality in normal weight individuals.

	(Poly)phenol Quartiles, OR (95% CI)				
	Q1	Q2	Q3	Q4	*P* for Trend
**Total (poly)phenols**	1	0.74 (0.45, 1.21)	0.52 (0.31, 0.89)	0.70 (0.39, 1.25)	0.060
**Total flavonoids**	1	0.60 (0.36, 1.01)	0.60 (0.36, 1.01)	0.66 (0.38, 1.13)	0.308
*Flavanols*	1	0.88 (0.44, 1.75)	0.66 (0.25, 1.71)	1.21 (0.39, 3.75)	0.571
Catechins	1	0.76 (0.45, 1.27)	0.84 (0.49, 1.42)	0.94 (0.57, 1.55)	0.869
*Flavonols*	1	0.86 (0.52, 1.45)	0.78 (0.47, 1.29)	1.03 (0.62, 1.71)	0.914
Quercetin	1	1.24 (0.71, 2.16)	1.55 (0.90, 2.68)	1.17 (0.63, 2.16)	0.827
Kaempferol	1	0.65 (0.39, 1.09)	0.68 (0.42, 1.10)	1.00 (0.53, 1.87)	0.228
*Flavanones*	1	1.32 (0.78, 2.23)	1.05 (0.61, 1.81)	0.88 (0.50, 1.52)	0.774
Hesperetin	1	1.24 (0.74, 2.09)	0.96 (0.56, 1.65)	0.87 (0.50, 1.50)	0.724
Naringenin	1	0.80 (0.48, 1.32)	0.51 (0.30, 0.85)	0.49 (0.28, 0.85)	0.016
*Flavones*	1	0.94 (0.56, 1.56)	0.64 (0.38, 1.07)	0.52 (0.30, 0.91)	0.037
Apigenin	1	0.78 (0.45, 1.34)	0.61 (0.34, 1.07)	0.67 (0.40, 1.13)	0.062
Luteolin	1	1.34 (0.82, 2.17)	0.77 (0.47, 1.28)	0.68 (0.39, 1.18)	0.106
*Anthocyanins*	1	1.04 (0.63, 1.69)	0.73 (0.44, 1.21)	0.59 (0.34, 1.03)	0.033
*Isoflavones*	1	0.99 (0.59, 1.63)	1.11 (0.66, 1.84)	1.00 (0.61, 1.63)	0.878
Daidzein	1	1.04 (0.63, 1.72)	1.14 (0.68, 1.91)	1.02 (0.62, 1.68)	0.803
Genistein	1	1.06 (0.64, 1.75)	1.02 (0.59, 1.76)	1.09 (0.66, 1.78)	0.992
Biochanin A	1	0.59 (0.33, 1.04)	0.79 (0.46, 1.37)	0.89 (0.49, 1.60)	0.823
**Phenolic acids**	1	1.22 (0.72, 2.05)	1.18 (0.70, 1.96)	0.79 (0.46, 1.35)	0.538
*Hydroxybenzoic acids*	1	1.24 (0.76, 2.00)	0.73 (0.42, 1.27)	1.15 (0.67, 1.97)	0.631
Vanillic acid	1	0.93 (0.55, 1.56)	1.18 (0.71, 1.97)	1.31 (0.74, 2.31)	0.816
*Hydroxycinnamic acids*	1	0.59 (0.36, 0.97)	0.60 (0.37, 0.97)	0.39 (0.22, 0.69)	0.049
Caffeic acid	1	1.22 (0.74, 1.99)	0.49 (0.29, 0.84)	0.86 (0.42, 1.79)	0.044
Cinnamic acid	1	0.84 (0.52, 1.35)	0.88 (0.52, 1.49)	0.73 (0.44, 1.21)	0.481
Ferulic acid	1	0.87 (0.51, 1.47)	0.92 (0.55, 1.54)	0.91 (0.52, 1.60)	0.312
**Stilbenes**	1	0.57 (0.33, 0.99)	0.79 (0.45, 1.39)	0.95 (0.43, 2.09)	0.553
**Lignans**	1	0.70 (0.42, 1.15)	0.45 (0.27, 0.77)	0.54 (0.31, 0.94)	0.040
Lariciresinol	1	0.79 (0.48, 1.30)	0.54 (0.32, 0.90)	0.61 (0.36, 1.05)	0.061
Matairesinol	1	0.70 (0.42, 1.16)	0.57 (0.35, 0.95)	0.50 (0.29, 0.87)	0.025
Pinoresinol	1	0.68 (0.41, 1.12)	0.47 (0.27, 0.80)	0.55 (0.32, 0.96)	0.030
Secoisolariciresinol	1	0.54 (0.33, 0.88)	0.54 (0.31, 0.92)	0.51 (0.29, 0.89)	0.047

Adjusted for total energy intake (continuous), body mass index (continuous), physical activity (low/medium/high), educational status (low/medium/high), occupational status (unemployed/low/medium/high), smoking status (current/former/never), alcohol consumption (none/moderate/regular), occurrence of hypertension, diabetes, dyslipidemias, cardiovascular disease, cancer (yes/no), and menopausal status (women only, yes/no).

**Table 4:** Odds ratios (ORs) and 95% confidence intervals (CIs) for the associations between (poly)phenol intake (total, main classes, subclasses and individual compounds) and adequate sleep quality in overweight/obese individuals.

	(Poly)phenol Quartiles, OR (95% CI)				
	Q1	Q2	Q3	Q4	*P* for Trend
**Total (poly)phenols**	1	1.64 (0.98, 2.73)	1.13 (0.82, 2.29)	1.57 (0.86, 2.83)	0.219
**Total flavonoids**	1	1.24 (0.73, 2.10)	1.50 (0.88, 2.55)	1.69 (0.93, 3.05)	0.075
*Flavanols*	1	1.37 (0.73, 2.56)	1.10 (0.46, 2.60)	1.37 (0.42, 4.46)	0.982
Catechins	1	0.88 (0.54, 1.43)	1.26 (0.74, 2.12)	1.31 (0.77, 2.24)	0.114
*Flavonols*	1	0.89 (0.53, 1.48)	1.16 (0.71, 1.91)	1.47 (0.90, 2.40)	0.183
Quercetin	1	1.55 (0.88, 2.73)	1.44 (0.83, 2.49)	1.46 (0.82, 2.60)	0.180
Kaempferol	1	0.90 (0.53, 1.53)	0.97 (0.57, 1.63)	0.93 (0.49, 1.76)	0.918
*Flavanones*	1	0.61 (0.37, 1.01)	1.00 (0.62, 1.61)	0.82 (0.51, 1.32)	0.847
Hesperetin	1	0.60 (0.36, 1.00)	0.99 (0.62, 1.59)	0.82 (0.51, 1.33)	0.803
Naringenin	1	0.86 (0.51, 1.43)	0.77 (0.46, 1.28)	0.99 (0.59, 1.65)	0.899
*Flavones*	1	1.00 (0.61, 1.64)	0.81 (0.50, 1.33)	0.89 (0.54, 1.47)	0.195
Apigenin	1	0.56 (0.33, 0.95)	0.61 (0.34, 1.07)	0.53 (0.31, 0.90)	0.555
Luteolin	1	0.80 (0.47, 1.34)	0.79 (0.48, 1.30)	0.88 (0.53, 1.45)	0.208
*Anthocyanins*	1	0.84 (0.48, 1.48)	1.17 (0.69, 1.97)	1.29 (0.73, 2.27)	0.155
*Isoflavones*	1	0.87 (0.53, 1.41)	0.96 (0.60, 1.53)	0.91 (0.54, 1.54)	0.349
Daidzein	1	0.81 (0.50, 1.32)	0.97 (0.60, 1.55)	0.84 (0.49, 1.44)	0.269
Genistein	1	0.80 (0.49, 1.30)	0.94 (0.58, 1.52)	0.85 (0.50, 1.43)	0.286
Biochanin A	1	1.02 (0.58, 1.79)	1.27 (0.73, 2.23)	1.45 (0.81, 2.60)	0.024
**Phenolic acids**	1	1.35 (0.83, 2.20)	1.38 (0.83, 2.28)	1.49 (0.90, 2.45)	0.079
*Hydroxybenzoic acids*	1	0.94 (0.58, 1.51)	1.41 (0.85, 2.35)	1.06 (0.64, 1.76)	0.308
Vanillic acid	1	1.04 (0.60, 1.80)	1.39 (0.80, 2.39)	1.14 (0.63, 2.04)	0.324
*Hydroxycinnamic acids*	1	1.28 (0.77, 2.12)	0.86 (0.50, 1.47)	1.03 (0.60, 1.78)	0.260
Caffeic acid	1	0.69 (0.40, 1.20)	0.90 (0.52, 1.52)	0.81 (0.40, 1.62)	0.860
Cinnamic acid	1	1.22 (0.75, 2.00)	0.99 (0.58, 1.69)	1.53 (0.93, 2.51)	0.195
Ferulic acid	1	0.61 (0.37, 1.00)	0.92 (0.58, 1.47)	0.72 (0.42, 1.22)	0.609
**Stilbenes**	1	0.99 (0.57, 1.69)	0.84 (0.46, 1.53)	0.91 (0.48, 1.72)	0.555
**Lignans**	1	1.15 (0.69, 1.91)	0.86 (0.52, 1.43)	1.23 (0.73, 2.07)	0.538
Lariciresinol	1	1.09 (0.66, 1.80)	0.90 (0.55, 1.47)	1.30 (0.77, 2.17)	0.396
Matairesinol	1	0.86 (0.52, 1.44)	0.84 (0.50, 1.41)	0.99 (0.58, 1.69)	0.864
Pinoresinol	1	1.13 (0.69, 1.84)	0.84 (0.50, 1.41)	1.11 (0.67, 1.86)	0.520
Secoisolariciresinol	1	1.02 (0.62, 1.70)	0.90 (0.54, 1.51)	1.37 (0.80, 2.33)	0.342

Adjusted for total energy intake (continuous), body mass index (continuous), physical activity (low/medium/high), educational status (low/medium/high), occupational status (unemployed/low/medium/high), smoking status (current/former/never), alcohol consumption (none/moderate/regular), occurrence of hypertension, diabetes, dyslipidemias, cardiovascular disease, cancer (yes/no), and menopausal status (women only, yes/no).
